# Longitudinal Assessment of Optical Quality and Intraocular Scattering Using the Double-Pass Instrument in Normal Eyes and Eyes with Short Tear Breakup Time

**DOI:** 10.1371/journal.pone.0082427

**Published:** 2013-12-06

**Authors:** Hidenaga Kobashi, Kazutaka Kamiya, Kyohei Yanome, Akihito Igarashi, Kimiya Shimizu

**Affiliations:** Department of Ophthalmology, University of Kitasato School of Medicine, Kanagawa, Japan.; Medical University Graz, Austria

## Abstract

**Purpose:**

To assess the longitudinal changes in optical quality including intraocular scattering in normal eyes and eyes with short tear breakup time (TBUT).

**Methods:**

We prospectively examined twenty eyes of 20 healthy subjects, and age-matched twenty eyes of 20 short TBUT subjects. The modulation transfer function (MTF) cutoff frequency, the Strehl ratio, and the objective scattering index (OSI) were quantitatively assessed using an Optical Quality Analysis System. We investigated the changes in these variables measured consecutively at the initial examination, 5, and 10 seconds without blinking. We also compared these variables in eyes with short TBUT with those in normal eyes.

**Results:**

No significant differences in the MTF cutoff frequency, Strehl ratio, or OSI were detected over a 10-second period in normal eyes. These variables also became significantly degraded even over a 5-second period in eyes with short TBUT (p<0.01). We found significant differences in these variables at 5 and 10 seconds (p<0.05), but none immediately after the blink between normal and short TBUT eyes.

**Conclusions:**

Optical quality including intraocular scattering deteriorated significantly with time in eyes with short TBUT, whereas we found significant differences over a 10-second period in normal eyes. Eyes with short TBUT showed greater deterioration in optical quality after the blink than normal eyes. The longitudinal assessment of optical quality may be effective in distinguishing eyes with short TBUT from normal eyes.

## Introduction

Dry eye can be classified as either aqueous tear-deficient dry eye or evaporative dry eye [1]. In mild cases of evaporative dry eye, the tear volume does not always decrease, and only shortening of the tear breakup time (TBUT) indicates a tear abnormality [2]. In a clinical setting, there are many borderline cases that fall between evaporative dry eyes and healthy eyes, in which short TBUT is found without dry eye symptoms, ocular surface damage, or tear deficiency [3]. In eyes with short TBUT, the tear film might begin to break up rapidly before the next blink during a period of suppressed blinking despite adequate tear volume on the ocular surface, which may result in symptoms of blurred vision, ocular fatigue, and discomfort. It has been reported that the shortness of TBUT has a large impact on vision [4], [5].

With regard to changes in optical quality and due to tear film dynamics, most studies have focused on the changes in corneal or ocular higher-order aberrations between blinks using corneal topographers or Shack-Hartmann wavefront sensors [6]–[13]. However, it has not been fully elucidated whether the tear film dynamics has any impact on intraocular scattering. The Optical Quality Analysis System (OQAS, Visiometrics, Terrassa, Spain) was designed for use in clinical practice to objectively determine the optical quality, including intraocular scattering, of the human eye using a double-pass technique. There have been only a few studies on tear film-related optical quality using this device in normal subjects or dry eye patients [14]–[16]. However, to our knowledge, there have so far been no clinical studies on detailed optical quality parameters, such as modulation transfer function (MTF) cutoff frequency and Strehl ratio, using the double-pass system. In addition, no study has addressed the changes in intraocular scattering as a measure of tear film quality in eyes with short TBUT, in which are borderline cases between dry eyes and healthy eyes. The purpose of this study is to prospectively evaluate the longitudinal changes in optical quality in normal eyes and eyes with short TBUT.

## Materials and Methods

### Subjects

Twenty eyes of 20 healthy volunteers (11 female, 9 male; average age, 31.2±3.8 years) and twenty eyes of 20 short TBUT subjects (12 female, 8 male; average age, 31.6±4.1 years) were enrolled in this study. All subjects were emmetropic or low-myopic with a visual acuity of 20/20 or better. One eye of each subject was chosen randomly for the measurement. The sample sizes in our study offered 87% statistical power at the 5% level to detect a 5-cycles/degree difference in MTF cutoff frequencies between the two groups, when the standard deviation (SD) of the mean difference was 5.0 cycles/degree. They also offered 97% statistical power at the 5% level to detect a 0.05-difference in the Strehl ratio between the two groups, when the SD of the mean difference was 0.04, and offered 94% statistical power at the 5% level to detect a 0.8-difference in the objective scattering index (OSI) between the two groups, when the SD of the mean difference was 0.7. Short TBUT eye was diagnosed in subjects when the TBUT values were equal to or shorter than 5 seconds, and the subjects had no dry eye-related symptoms with no positive fluorescein staining or scores less than 3 points. The standard TBUT measurement was performed. After 1% fluorescein dye was instilled into the conjunctival sac, the interval between the last complete blink and the appearance of the first corneal black spot in the stained tear film was measured 3 times and the mean value of the measurements was calculated. The fluorescein score was assessed with a 1% fluorescein solution using the 0-9 scoring system as described by Shimmura et al [17]. The staining of the superior cornea, mid-cornea, and inferior cornea was graded on a scale of 0 (no staining) to 3 (intense staining) using a slit-lamp microscope. Natural tear volume was measured using Schirmer I test, in which the extent of tear flow down a piece of filter paper inserted into the lateral part of the inferior fornix of the eye over a 5-minute period without anesthetic drops. Any history of previous ocular surgery, ocular trauma, contact lens use, punctual occlusion or diathermy, eyes with dry eye symptoms, or eye disease including active inflammation of the eye, was excluded from the study. The study was approved by the Institutional Review Board at Kitasato University School of Medicine, and followed the tenets of the Declaration of Helsinki. Written informed consent was obtained from all patients after explanation of the nature and possible consequences of the study.

### Optical Quality Measurement

We measured the optical quality parameters of the eye, such as the MTF cutoff frequency, the Strehl ratio, and the OSI, using the OQAS for a 4.0-mm pupil. Near-infrared light consisting of a laser diode (wavelength, 780 nm) is used because it is more comfortable for the subject and provides retinal image quality estimates that are comparable to those obtained with visible light. The MTF cutoff in the double-pass system is the frequency at which the MTF reaches a value of 0.01. Because the point spread function (PSF) images recorded by the double-pass instrument can be affected by high-frequency noise, which is inherent in the use of cameras, the frequency for very small MTF values may become unstable, potentially leading to artifacts. To avoid this problem, the device uses an MTF threshold value of 0.01, which corresponds to 1% contrast. Thus, the MTF cutoff frequency in this article refers to the frequency up to which the eye can focus an object on the retina with a significant 1% contrast. The Strehl ratio is an expression of the ratio of the central maximum of the illuminance of the PSF in the aberrated eye to the central maximum that would be found in a corresponding aberration-free system. It is the measure of the fractional drop in the peak of the PSF as a function of the wavefront error. The OSI is an objective evaluation of intraocular scattered light. The index is calculated by evaluating the amount of light outside the double-pass retinal intensity PSF image in relation to the amount of light on the center. In the particular case of the instrument OQAS, the central area selected was a circle of a radius of 1 minute of an arc, while the peripheral zone was a ring set between 12 and 20 minutes of arc [18].

### Experimental Procedure

The subject was instructed to blink three or four times and fixate on a distant image created by the double-pass system with the eyes kept wide open for as long as possible. During this period without blinking, 3 consecutive images were captured with the double-pass system. The first was taken immediately after the blink (nominally, the initial examination) and the others 5 and 10 seconds later, the timing accuracy for the latter being ± 1 second. The study assessed the optical quality parameters at the initial examination, 5, and 10 seconds after the blink. We also calculated the rate of change in these parameters obtained after 10 seconds without blinking according to the following equation:

Change rate (%)  =  (Value at 10 seconds - value at initial examination)/(value at initial examination)

The manifest refractive error of the subjects was fully corrected during these measurements; the spherical error was automatically corrected by the double-pass system, and the cylindrical error was corrected with an external lens, because the uncorrected refractive error directly affects the optical outcome of the system. In all eyes, the pupil diameter, provided by this device from an image of an additional video camera that allowed pupil alignment, was more than 4.0 mm. The room illumination was kept low (approximately 25 lux) during testing. The tests were run in a controlled temperature (22±3°C) and humidity (40±4%).

For each subject, between 2 to 4 series of double-pass images were recorded. Only well-recorded double-pass images taken between blinks were analyzed, which is in line with Benito et al. [15]. It has been demonstrated that this device has excellent repeatability of measurement [19], [20]. To confirm the repeatability of the measurements, the measurements with this device were made in 20 short-TBUT eyes at 5 seconds after the blink at the same time of day on two days. We evaluated the repeatability of the two measurements as described previously using Bland-Altman plots [21].

### Statistical Analysis

All statistical analyses were performed using SPSS (SPSS Inc, Chicago, IL, USA). Repeated-measures analysis of variance (ANOVA), followed by the Dunnett post hoc test for multiple comparisons, was used to compare the differences with those at 5 and 10 seconds after a blink. Normality of all data samples was first checked by the Kolmogorov-Smirnov test. Since the use of parametric statistics was not possible, the Mann-Whitney *U* test was also used to compare data between the two groups. The data were analyzed with the Fisher’s exact test for the percentage of female. The results are expressed as mean ± standard deviation, and a value of p<0.05 was considered statistically significant.

## Results

The demographic data of the study population are shown in Table 1. There was no significant differences in terms of age (p = 0.73, Mann-Whitney *U* test), gender (p = 1.00), fluorescein score (p = 0.38), and Schirmer I test (p = 0.08), between the two groups. The TBUT was significantly decreased in the short TBUT eye group in comparison with the normal eye group (p<0.001).

**Table 1:** Demographics of normal eyes and eyes with short TBUT.

	Normal eye (n = 20)	Short TBUT eye (n = 20)	P-value
Age (years)	31.2±3.8 (range, 20 to 38 years)	31.6±4.1 (range, 25 to 37 years)	0.73
Gender (% female)	55%	60%	1.00
TBUT (s)	9.1±2.4 (range, 6 to 14 s)	3.0±1.1 (range, 1 to 5 s)	<0.001
Fluorescein score (point)	0.1±0.3 (range, 0 to 1)	0.2±0.4 (range, 0 to 1)	0.38
Schirmer I test (mm)	18.1±4.3 (range, 10 to 24 mm)	16.0±4.0 (range, 10 to 23 mm)	0.08

TBUT = tear breakup time.

**Figure 1:**
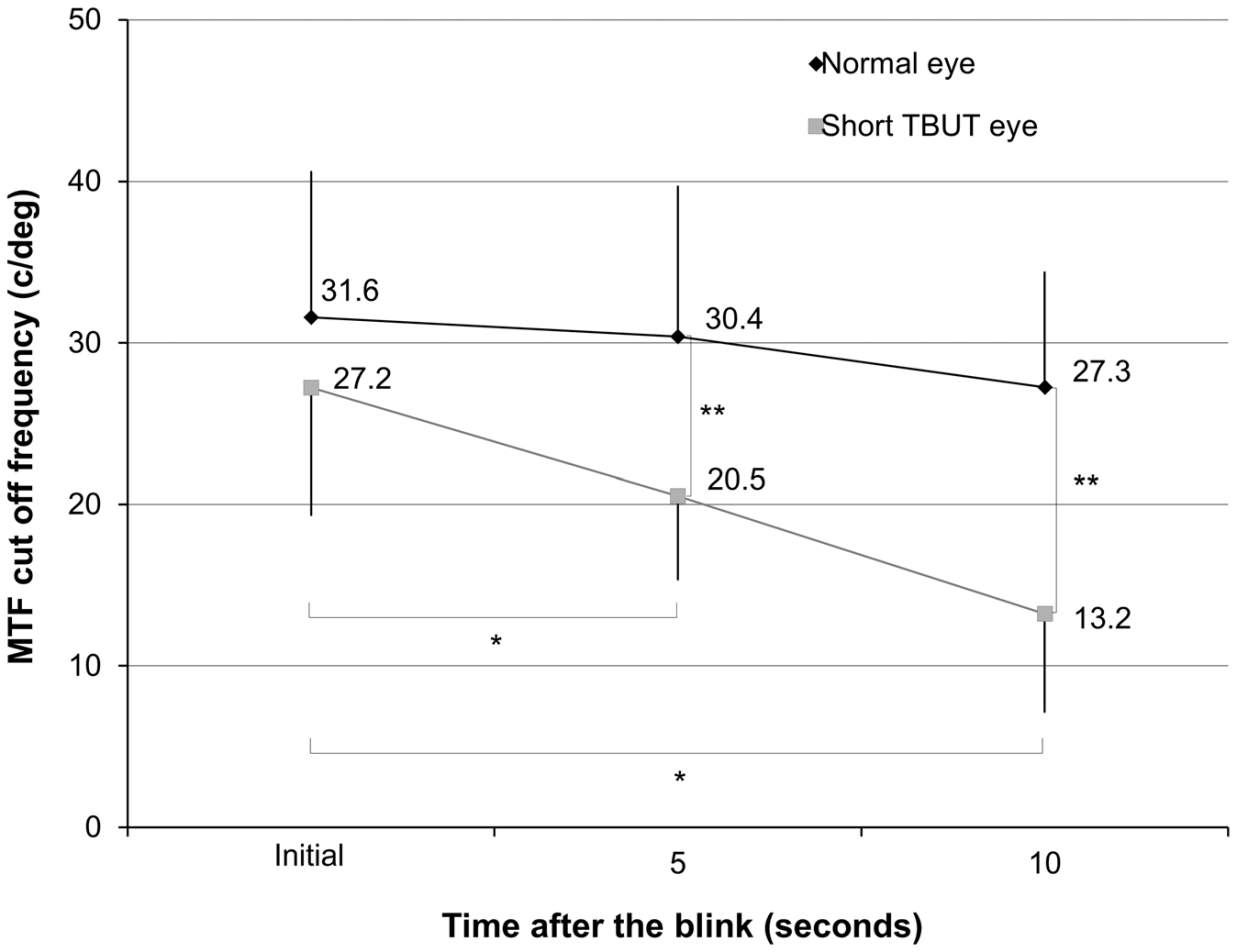
Changes in MTF cutoff frequency after the blink in normal eyes and eyes with short TBUT. * represents significant difference between time after the blink (p<0.05). ** also represents significant difference between both groups (p<0.05). MTF = modulation transfer function, TBUT = tear breakup time.

The mean MTF cutoff frequency at the initial examination, 5, and 10 seconds after the blink were 31.6±9.0, 30.4±9.3, and 27.3±7.1 cycles/degree, respectively, in the normal eye group (Figure 1). The ANOVA showed no significant difference between time points (p = 0.26). Corresponding values were 27.2±7.9, 20.5±5.2, and 13.2±6.1 cycles/degree, respectively, in the short TBUT eye group. The ANOVA showed a significant difference between time points (p<0.001). Dunnett post hoc test demonstrated a significant difference between measurements made at the initial examination and at 5 seconds (p = 0.003), and those made at the initial examination and at 10 seconds (p<0.001). We found significant differences in the MTF cutoff frequency at 5 and 10 seconds after the blink (p<0.001), but no significant differences at the initial examination between the normal and short TBUT groups (p = 0.12). The mean rate of changes in MTF cutoff frequency within 10 seconds in eyes with normal and those with short TBUT were –9.3±27.5% and –47.5±31.7%, respectively (p<0.001).

**Figure 2:**
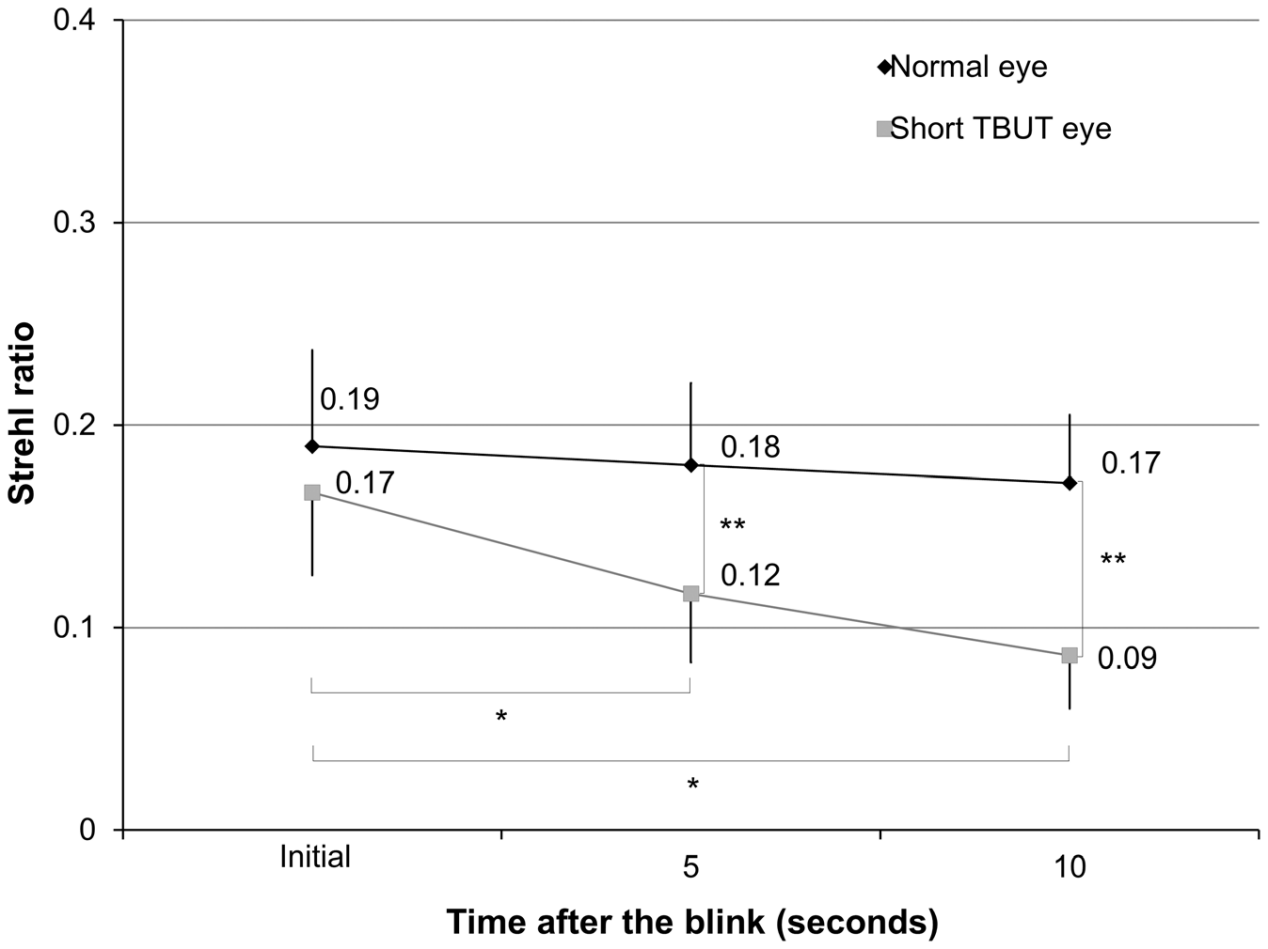
Changes in Strehl ratio after the blink in normal eyes and eyes with short TBUT. * represents significant difference between time after the blink (p<0.05). ** also represents significant difference between both groups (p<0.05). TBUT = tear breakup time.

The mean Strehl ratio at the initial examination, 5, and 10 seconds after the blink were 0.19±0.05, 0.18±0.04, and 0.17±0.03, respectively, in the normal eye group (Figure 2). The ANOVA showed no significant difference between time points (p = 0.38). Corresponding values were 0.17±0.04, 0.12±0.03, and 0.09±0.03, respectively, in the short TBUT eye group. The ANOVA showed a significant difference between time points (p<0.001). Dunnett post hoc test demonstrated a significant difference between measurements made at the initial examination and at 5 seconds (p<0.001), and those made at the initial examination and at 10 seconds (p<0.001). We found significant differences in the Strehl ratio at 5 and 10 seconds after the blink (p<0.001), but no significant differences at the initial examination between the two groups (p = 0.07). The mean rate of changes in the Strehl ratio within 10 seconds in eyes with normal and those with short TBUT were –5.8±21.0% and –46.1±17.7%, respectively (p<0.001).

**Figure 3:**
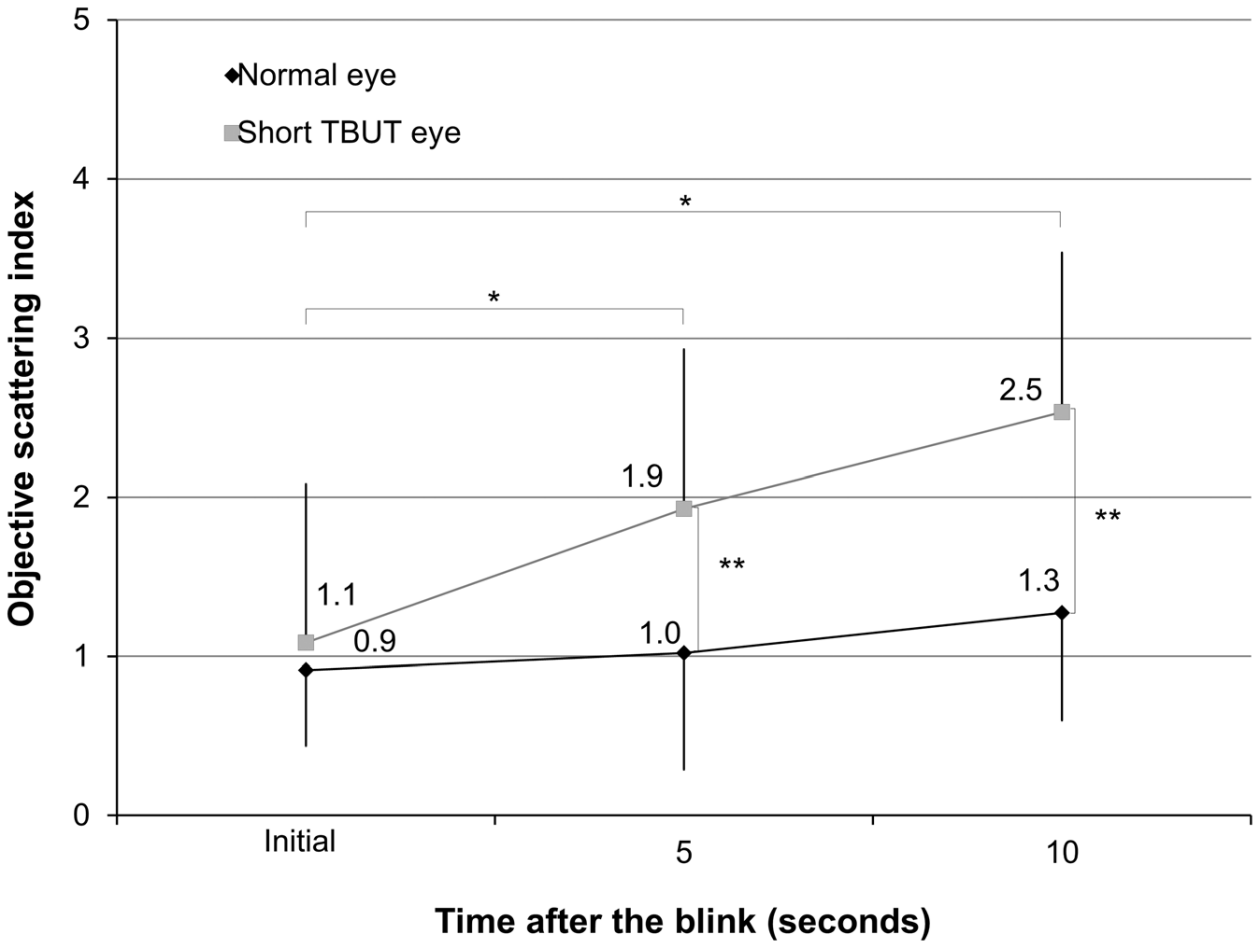
Changes in OSI after the blink in normal eyes and eyes with short TBUT. * represents significant difference between time after the blink (p<0.05). ** also represents significant difference between both groups (p<0.05). OSI = objective scattering index, TBUT = tear breakup time.

The mean OSI at the initial examination, 5, and 10 seconds after the blink were 0.9±0.5, 1.0±0.7, and 1.3±0.7, respectively, in the normal eye group (Figure 3). The ANOVA showed no significant difference between time points (p = 0.71). Corresponding values were 1.1±0.4, 1.9±0.8, and 2.5±0.7, respectively, in the short TBUT eye group. The ANOVA showed a significant difference between time points (p<0.001). Dunnett post hoc test demonstrated a significant difference between measurements made at the initial examination and at 5 seconds (p<0.001), and those made at the initial examination and at 10 seconds (p<0.001). We found significant differences in the OSI at 5 and 10 seconds after the blink (p<0.001), but no significant differences at the initial examination between the two groups (p = 0.17). The mean rates of changes in OSI within 10 seconds in eyes with normal and those with short TBUT were 46.8±39.4% and 165.9±114.2%, respectively (p<0.001).

**Figure 4:**
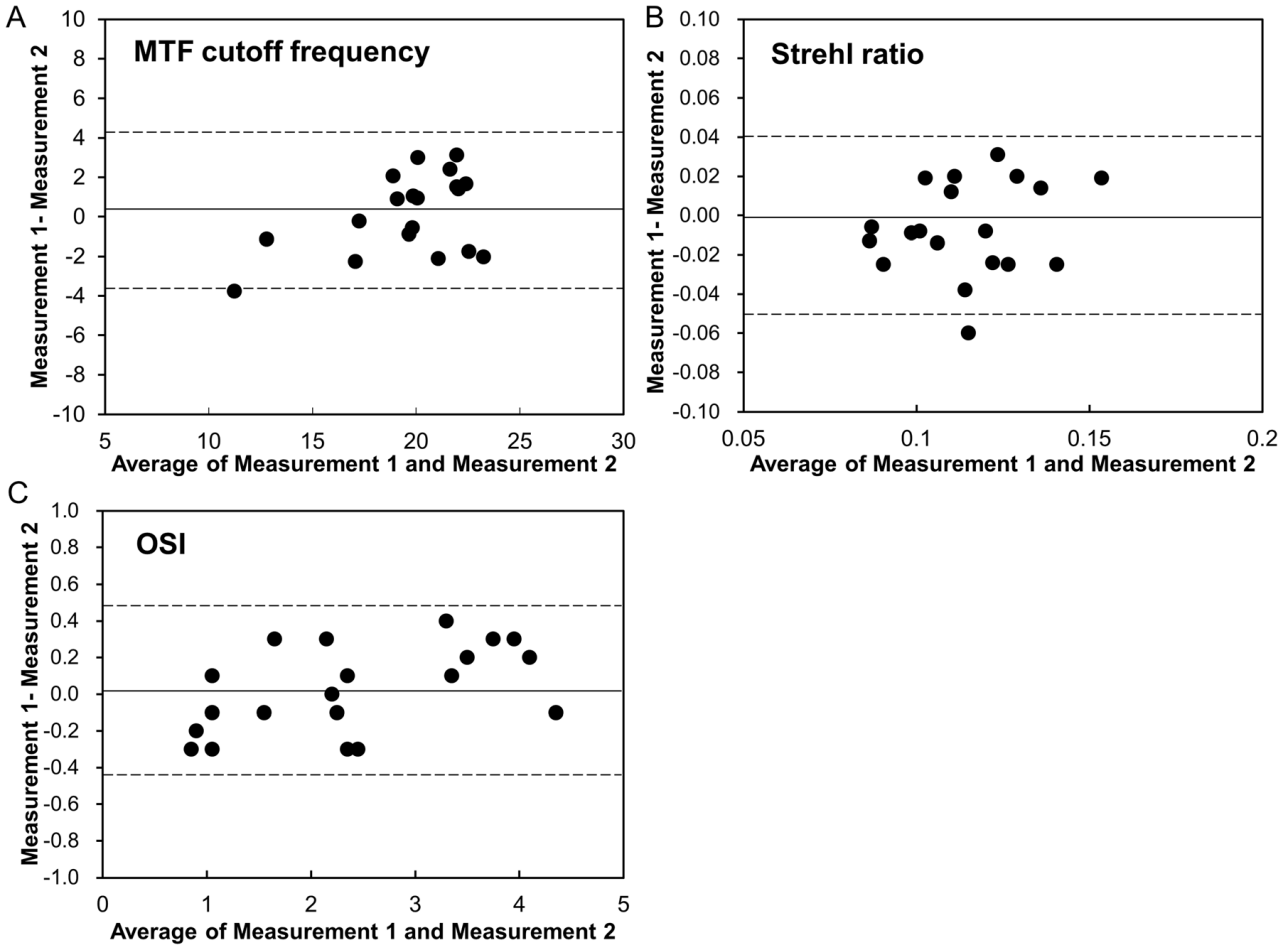
Bland-Altman plots represent the difference between two measurements divided by the mean of these measurements. A, MTF cutoff frequency. B, Strehl ratio. C, OSI. The solid lines represent mean differences between 2 measurements of MTF cutoff frequency and Strehl ratio; dotted lines are the upper and lower borders of the 95% limit of agreement (mean difference ± 1.96 multiplied by standard deviation of the mean difference). MTF = modulation transfer function, OSI = objective scattering index.

Bland-Altman plots indicate that the mean difference between two measurements with this device (± 95% limits of agreement; LoA) was 0.31±2.03 cycles/degree (–3.66 to 4.28 cycles/degree) for MTF cut-off frequency, –0.01±0.02 (–0.05 to 0.04) for Strehl ratio, and 0.03±0.24 (–0.44 to 0.49) for OSI (Figure 4).

## Discussion

In the current study, we demonstrated that the MTF cutoff frequency and the Strehl ratio decreased significantly, and the OSI increased significantly, with time after a blink, in eyes with short TBUT. In contrast, we found no significant differences in these variables over a 10-second period in normal eyes. These results indicate that, in eyes with short TBUT, the optical quality of the eye may have deteriorated with time after a blink.

With regard to normal eyes, no significant differences in optical quality, including intraocular scattering, were detected over a 10-second period. Diaz-Valle et al. [16] reported that no significant differences were found in intraocular scattering over a 20-second period, which was consistent with our findings. To our knowledge, this is the first published study to assess the detailed optical quality parameters using the double-pass method, such as the MTF cutoff frequency and the Strehl ratio, which are considered to have a large impact on visual performance. Montés-Micó et al. [14] found that optical performance in normal subjects gradually deteriorated 6 seconds after the blink, which was inconsistent with our present findings. However, they did not provide a detailed analysis of these optical quality parameters, such as the MTF cutoff frequency or the Strehl ratio.

With regard to short TBUT eyes, the optical quality including the intraocular scattering significantly degraded even over 5-second period. We also found significant differences in these variables at 5 and 10 seconds after the blink, but no significant differences immediately after the blink between the normal and short TBUT groups. Our findings of short TBUT eyes using the double-pass method are in line with those in previous report using the Shack-Hartmann wavefront sensor [4]. As far as we can ascertain, this is also the first study to assess the longitudinal changes in intraocular scattering as a measure of tear film quality in eyes with short TBUT. Tear film instability expressed as short TBUT (< 5 seconds) may be accepted as a component of dry eye [22], [23]. Eyes with short TBUT may be diagnosed as early-stage or borderline cases of evaporative dry eye despite normal Schirmer test values and negative ocular surface damage. The impact of the tear film on the quality of the retinal image depends greatly on the homogeneity of the tear pellicle [15]. In eyes with short TBUT, the loss of homogeneity in the tear film may cause significant deterioration in the retinal image quality including wavefront aberrations and light scattering. Therefore, we believe that the longitudinal assessment of optical quality is effective in distinguishing eyes with short TBUT from normal eyes. This promising approach may allow early detection and follow-up of tear film-related patient complications.

In the current study, we also demonstrated that eyes with short TBUT showed significantly higher change rates in the optical quality parameters than did those with normal subjects, indicating that the degradation of visual performance under natural viewing conditions may occur more often in eyes with short TBUT than in those of normal subjects. Our results are in line with those reported in dry eye patients by Diaz-Valle et al. [16] It is suggested that an unstable tear film may play a role in enhancing ocular scattering in eyes with short TBUT, and that these change rates are potential indicators for differentiating short-TBUT eyes from normal eyes. A more detailed analysis should be performed to determine the exact role of the change rate of optical quality in eyes with short TBUT.

It is important to assess the repeatability of the measurements with this instrument in order to confirm the applicability of the data. It has been demonstrated that the device has good repeatability [19], [20], and that the realignment of the eyes does not impose any additional variation on the measurements [19], [20]. As shown in Figure 4, we confirmed the good repeatability of the measurements in the current study, as evidenced by the narrow 95% LoA. Hence, we believe that this device offers reasonable repeatability in the clinical evaluation of the optical quality of the eye.

There are at least two limitations to this study. First, we assessed these optical parameters only for 4.0-mm pupils. Considering that all subjects had few fluorescein scores localized in the inferior cornea, we assume that there is no significant clinical impact on optical quality parameters for a 4.0-mm pupil. Further studies are required to determine optical quality parameters in the tear film dynamics under several pupil size conditions. Second, in our study using the double-pass technique, we measured optical quality at 5-second intervals over 10 seconds without blinking, whereas recent studies were performed every 0.5-second intervals over 20 seconds [15], [16]. However, in order to simply compare short TBUT subjects with normal subjects, we evaluated 3 measurement points (initial examination, 5, and 10 seconds after the blink). Moreover, we assessed the detailed optical quality parameters, such as the MTF cutoff frequency and the Strehl ratio, whereas previous studies evaluated only OSI [15], [16]. We are currently conducting a new study on serial measurements during the 20 seconds after blinking.

In conclusion, our results demonstrated that optical quality including the intraocular scattering deteriorated significantly with time in eyes with short TBUT, whereas we found no significant differences over a 10-second period in normal eyes. Our results also showed that eyes with short TBUT had a greater deterioration in optical quality after the blink than normal eyes. It is suggested that the longitudinal assessment of optical quality is effective in distinguishing short TBUT eyes from normal eyes.
